# Sustainability and minimization of carbon footprint in ophthalmology: what can we change in the outpatient clinic routine?

**DOI:** 10.1038/s41433-025-03885-0

**Published:** 2025-06-18

**Authors:** Zeynep Akgun, Melis Palamar

**Affiliations:** 1 Van Research and Training Hospital, Department of Ophthalmology, Van, Türkiye; 2 Ege University Faculty of Medicine, Department of Ophthalmology, Izmir, Türkiye

**Keywords:** Health care economics, Epidemiology

## Abstract

Ophthalmological care has significant potential to consume energy, utilize resources, and generate waste, thereby contributing to a substantial portion of greenhouse gas emissions. Although awareness is increasing, many clinicians still lack knowledge about the actions that can be taken to address climate change. The literature about sustainability in ophthalmology has focused on surgery and the operating room. In this review, it was aimed to explore what can be accomplished in outpatient clinics, which make up a significant portion of the daily routine. Outpatient clinic services contribute significantly to carbon emissions. Measures to reduce this impact include redesigning outpatient clinics, using multi-purpose tools and equipment that can be properly disinfected and sterilized rather than relying on single-use items, minimizing unnecessary referrals and visits, and incorporating artificial intelligence and telemedicine into ophthalmological care.

## Introduction

*“The Earth is a fine place and worth fighting for.”-Ernest Hemingway*

According to the World Health Organization (WHO), climate change is one of the greatest threats to global health in the 21^st^ century [1]. Despite several actions, global greenhouse gas (GHG) emissions continue to rise [2]. The uncontrolled use of resources, particularly over the past 50 years, has made human existence unsustainable. A clear example of this is Earth Overshoot Day, defined as the day when global annual resource consumption exceeds the Earth’s capacity to regenerate for a given year. This date was December 29 in 1970, and it shifted to July 29 in 2019 [3]. With the increase in resource consumption after the pandemic, it dropped to July 27 in 2023 [4].

Carbon footprint refers to the total amount of GHG produced directly or indirectly by human activities and is expressed as a carbon dioxide equivalent (CO2eq) [5]. Due to their broad scope and specific requirements, healthcare services have a significant potential to consume energy, use resources, and generate waste. Consequently, they play an essential role in contributing to the climate crisis. According to data from the World Bank and the WHO, the healthcare sector, which employed 65.1 million people globally as of 2020, is responsible for about 4–5% of global CO2eq emissions [6]. When examined by country, healthcare services account for 10.4% of CO2eq emissions in the United States (US), 7.3% in Australia, 5% in Canada, 6% in Japan, and 4% in the United Kingdom (UK) [7–9]. Although there are no direct statistics on the share of healthcare services in total CO2eq emissions in Türkiye, it is estimated to be correlated with economic growth and an increase in healthcare expenditures [10].

The growing population and increasing life expectancy are contributing to a larger carbon footprint in healthcare. Therefore, it is crucial to swiftly adopt more sustainable practices in medicine. Recently, international efforts to assess and reduce the carbon footprint of the healthcare sector have gained momentum. As of May 2024, 82 countries have committed to providing more sustainable healthcare services through the Alliance for Transformative Action on Climate Change and Health, which is led by WHO [11]. Additionally, the Lancet Commission on Health and Climate is working to enhance the sustainability of healthcare services [12].

Ophthalmological care is a significant component of the healthcare burden. It encompasses patients of all ages and covers common conditions like refractive errors, cataracts, and glaucoma. Ophthalmology involves a high volume of consultations, procedures, surgeries, and chronic diseases such as age-related macular degeneration and glaucoma [13]. It was the highest-volume specialty, accounting for 8.1% of outpatient visits in the UK during 2018–19 [14]. The growing population, increasing life expectancy, and expanding care and surgical interventions will likely raise the proportion of ophthalmology within healthcare. Therefore, it is crucial to implement sustainability in ophthalmology practices while maintaining a high standard of care. EyeSustain (website-eyesustain.org) is an organization that provides education and research on sustainability in ophthalmology. It is supported by 50 global ophthalmology societies, including the European Society of Cataract and Refractive Surgery, the American Society of Cataract and Refractive Surgery, and the American Academy of Ophthalmology. The website contains a continuously updated collection of studies, articles, lectures, and position papers. It aims to increase ophthalmologists’ awareness, promote sustainable practices, and ensure that these practices can be implemented throughout the broader healthcare system [6, 15].

Cataract surgery is one of the highest-volume procedures, not only in ophthalmology but also across all medical specialties globally. Consequently, efforts to enhance the sustainability of cataract surgery and the operating room have gained significant importance in the current literature [16–18]. However, the outpatient clinic is a vital component of daily practice that experiences considerable patient turnover. Often, this leads to significant waste generation and energy consumption without awareness.

Despite the growing awareness, many clinicians still feel uncertain about how to address climate change. In this review, we aim to explore what can be done in outpatient eye examinations to promote sustainability and reduce the carbon footprint.

## Literature review

A literature search was conducted on PubMed and Google Scholar databases regarding sustainability and carbon footprint reduction in healthcare delivery. The search utilized the keywords “sustainability”, “carbon footprint”, “greenhouse gases”, “telemedicine”, “teleophthalmology”, “climate change”, “ophthalmology”, “eye health”, “ophthalmic surgery”, “cataract surgery”, “ outpatient clinic practice”, “examination tools”, “single-use “, “multi-use”, “medicines”, “packaging”, “travel” and “artificial intelligence”. No limitations regarding time, language, or geography were applied. Article titles and abstracts were scanned, and potentially relevant articles were retrieved for full-text evaluation. For articles not published in English or without translations, only English abstracts were used. Since the review focused on ophthalmological examinations, studies on this subject were prioritized. In addition to academic databases, Ministry of Health reports were analyzed to evaluate the practices and statistics in our country.

## Five fundamental principles of sustainability

Sustainability strategies can be summarized through the 5R’s (Reduce, Reuse, Recycle, Rethink, and Research) rule. Energy consumption can be reduced by turning off unused lights and equipment in operating rooms [19]. At Providence St. Peter Hospital in Washington, the operating room’s ventilation system was decreased by 60% during idle times, resulting in a significant reduction in energy consumption [20]. Kwakye et al. [21]. reported that switching from disposable to reusable surgical gowns reduced carbon dioxide waste by 23,000 kg over a 12-month period and saved $60,000. The separation and recycling of waste represent another pillar of sustainability. While concerns about the costs and carbon emissions related to collection, separation, and reprocessing continue, a study published in 2015 by McGain et al. [22] assessed the efficiency and cost of recycling operating room waste. Consequently, approximately 20% to 25% of this waste could be effectively recycled without compromising infection control or financial constraints. Another study analyzed waste generated after cataract surgery, finding an average of 0.827 kg per case. Approximately 57% of this was clinical waste, 38% was general waste, and 6% was sharp waste. It was determined that 51% of the general waste was recyclable [23]. Regarding the Rethink and Research steps, it is noteworthy that most of the literature focuses on operating rooms and surgical procedures [13, 20]. However, research on what can be achieved in outpatient clinics and their outcomes is quite limited.

## What modifications can be made in outpatient clinics?

### Inspiration from COVID-19 measures: green outpatient clinics

A notable study on applying sustainability principles in outpatient clinics was conducted by Ramesh et al. at Mahatma Eye Hospital in India [24]. The research team was inspired by the isolation measures during the COVID-19 pandemic. During the pandemic, a waiting room of approximately 450-square-foot was converted into a compact 256-square-foot examination room where all the essential devices were centrally located. The unit included two slit lamps, two computers, an automatic refractometer, a lensometer, a confocal fundus capture device, spectral-domain optical coherence tomography, optical biometry, manual keratometry, corneal topography, and non-contact tonometry, all linked through a local network. The patient walked around the room to complete the examination. Over two years, the authors utilized this unit. They found that its introduction significantly reduced the hospital’s total electricity consumption, decreasing from roughly 22,000 kilowatts per month to 13,000 kilowatts per month. They explained this result by pointing out that many ophthalmic devices require air conditioning for cooling, and placing them in different rooms causes GHG emissions from multiple air conditioners and a large amount of electricity consumption. Another advantage of the unit is that consolidating all the devices in one room reduces the number of technicians required and, consequently, lowers costs. Additionally, utilizing the local network, data from all the devices was collected on a single computer. This minimized paper usage and physical file storage while enhancing efficiency in terms of time and labor [25].

### Single or multiuse examination tools: pros and cons

There is an increasing trend in the use of single-use tools during outpatient clinic examinations. The main reason of this trend is the increased risk of transmitting diseases such as keratoconjunctivitis, herpetic eye disease, and prion disease due to inadequately disinfected equipment [26–29]. Single-use materials theoretically eliminate the risk of cross-contamination between patients and provide a safer alternative by eliminating the need for disinfection [30, 31]. However, these materials present some handicaps, such as high costs and significant carbon footprints. Park et al. [32] examined the costs and carbon footprints associated with applanation tonometry and gonioscopy, which are vital tools for diagnosing and treating glaucoma. They found that single-use tonometers incur an average annual cost of $70,282 and generate roughly 100.8 kg of plastic waste, while single-use gonioscopy lenses cost about $9040 per year and produce ~8.8 kg of plastic waste. In brief, reusable tonometers and gonioscopy lenses can be used safely, are environmentally friendly, and cost-effectively without causing significant contamination after disinfection with 70% isopropyl alcohol or diluted bleach.

### Single or multiuse examination drops: pros and cons

One of the most significant factors contributing to GHG emissions in ophthalmology is using single-use eye drops. Berkowitz et al. [33] assessed the cost of a multiuse dilating drop protocol before surgery and injection. They found a 97.1% reduction in the number of bottles and a cost savings of $240,000 over five years. Rahemtulla et al. [34] also compared the costs of multiuse and single-use dilating drops, finding that the number of bottles and costs were statistically significantly higher in the single-use group (*p* < 0.001 for both). As previously mentioned, the primary concern prompting ophthalmologists to utilize single-use materials is the risk of contamination. A controversial study on this topic was conducted by Haripriya et al. at Aravind Eye Hospital in India [35]. The research team used multiuse drops after cataract surgery and discovered that the endophthalmitis rate was lower (0.005%) than the US data (0.006%), where personalized drop therapy was applied postoperatively.

There are also two noteworthy UK studies regarding the costs and risks associated with the contamination of drops frequently used in outpatient clinics. Rautenbach et al. [36] investigated the risk of contamination from the multiple use of a single-use preservative-free proxymetacaine (0.5%) combined with fluorescein (0.25%). Normal conjunctival and lid flora, primarily *coagulase-negative Staphylococci* and *Corynebacterium spp*. were detected in 17% of the samples. However, they also found that switching to a single-use procedure would lead to an estimated threefold increase in the annual budget. In another study, Somner et al. [37] investigated single-use proxymetacaine (0.5%) combined with fluorescein (0.25%), phenylephrine (10%), and tropicamide (1%) drops. The contamination rate per drop application was 2.5%. The risk of cross-contamination with *coagulase-negative Staphylococci* is 1:400 and 1:80, respectively, when the bottle is used once or six times. The cost of reducing this risk to zero ranges from €2.75 to €4.6 million annually. Additionally, the single-use strategy generates 6.85 to 11.42 tons more paper waste and 12.69–21.15 tons more plastic waste. As a result, reducing contamination has financial and environmental costs. Conversely, exposure to *coagulase-negative Staphylococci* is not always associated with infection. With proper usage and staff training, multi-use eye drops can be safe, cost-effective, and more environmentally friendly.

### Brochures and packaging

Brochures and general packaging represent a significant portion of the waste generated during outpatient clinic visits. It is estimated that they account for 15% of healthcare’s GHG emissions. These materials, including drug prospectuses and information pamphlets about chronic diseases such as glaucoma, age-related macular degeneration (AMD), blepharitis, or keratoconus, as well as user manuals for items like contact lenses, lens solutions, and eyelash cleaning solutions, are often written in various languages, span multiple pages, and are rarely used [38, 39]. Substituting them with QR codes may be more eco-friendly [39]. This change will decrease packaging volume and shipping needs, thus aiding in the reduction of costs and environmental harm. However, many eye diseases, especially chronic problems such as glaucoma and AMD, are more prevalent in older patients who may be digitally inexperienced. It is important to note that the use of QR codes in this population will be limited due to poor vision and lack of digital experience [40].

### A different perspective on the debate regarding whether OCTA can replace FA

In 2019, Reynolds et al. [41] evaluated this controversial issue from a different perspective. They compared the carbon footprints of optical coherence tomography angiography (OCTA), a new technology used to detect retinal vascular diseases, with those of traditional fluorescein angiography (FA). FA can rarely be performed on the same day and usually requires a second visit, while OCTA can be conducted concurrently. It was found that FA emitted more GHG compared to OCTA, including 40.17 KgCO2eq from building costs, 33.03 KgCO2eq from personnel travel, 4.27 KgCO2eq from patient travel, 1.01 KgCO2eq from medicine, 1.41 KgCO2eq from medical devices, 1.24 KgCO2eq from waste. In addition to its many advantages, OCTA also helps reduce the carbon footprint of ophthalmology by decreasing the use of FA.

### Reorganizing of outpatient clinic services

Ophthalmology is one of the busiest specialties worldwide, and patient volumes are expected to increase gradually. A study conducted in the UK suggests that the number of patients requiring cataract surgery is projected to rise by 50% over the next 20 years, while the number of patients followed for glaucoma is anticipated to grow by 44% in the next 15 years [42]. Inappropriate referrals to outpatient ophthalmology services are estimated to range from 20% to 65% in the UK [43, 44]. Although there is no data on inappropriate referrals in Türkiye, the latest published data (Health Ministry, Health Statistics Yearbook, 2022) indicates that ophthalmology is among the three areas with the highest number of outpatient services [45]. This situation results in unnecessary costs, capacity issues, carbon emissions from travel, and waste production. There is a need for reorganizations to prevent unnecessary referrals and to implement population-based monitoring of chronic diseases like glaucoma and AMD.

### Minimizing carbon footprint from travel: telemedicine/teleophthalmology

Telemedicine, which gained widespread use during the COVID-19 pandemic and has continued to grow since then, offers significant benefits in terms of saving time, energy, and labor [46]. According to data from the UK National Health Service, staff commuting and patient travel make up 10% of healthcare emissions [47]. Purohit et al. [48] found that by preventing unnecessary patient transportation to health centers through telemedicine, the carbon footprint is reduced by between 0.7 and 372 kg of CO2eq per consultation. Lathan et al. [49] reported that avoiding unnecessary transportation through telemedicine is equivalent to planting 6.9 trees for each patient. Additionally, Sharafeldin et al. [50] noted that telemedicine is cost-effective for managing chronic pathologies such as diabetic retinopathy and glaucoma. While teleophthalmology will never replace precise visual acuity assessments and comprehensive clinical examinations, it does facilitate the remote delivery of appropriate services, improve access in geographically isolated areas, and reduce the carbon footprint.

### Incorporating AI and innovations in ophthalmological care

The integration of artificial intelligence (AI) into ophthalmic imaging, such as digital fundus photography and visual fields, has proven effective in screening and diagnosing prevalent sight-threatening diseases, including diabetic retinopathy, glaucoma, AMD, and retinopathy of premature [51–55]. Additionally, several home screening devices have been introduced to assess visual acuity, visual field, and intraocular pressure in conditions such as diabetic retinopathy, AMD, and glaucoma. ForeseeHome™, which conducts hyperacute perimetry for AMD monitoring, myVisiontrack™, which provides pattern separation and hyperacute perimetry for both AMD and diabetic retinopathy, and Alleye™, which can differentiate between neovascular and non-neovascular AMD, have received FDA approval [56–59]. AI-based systems have some disadvantages, including the need for extra costs and hardware, the ability to screen only the diseases for which they are programmed, the requirement for different deep learning systems for different diseases, and the potential for false positives and negatives [60, 61]. However, integrating these technologies into current monitoring programs also supports telemedicine by optimizing workforce efficiency and reducing carbon footprint.

### A brief overview: What is the total carbon footprint for a routine day at an outpatient clinic?

Di Felici et al. [62] calculated the daily carbon footprint of an ophthalmology consultation clinic in 2024. They included the carbon footprint of energy consumption, patient travel, staff travel, pharmaceutical and medical device purchases, computer hardware, biomedical equipment/examination materials, and waste in their calculations. Data were collected for each parameter through hospital statistics or surveys. The “Bilan Carbone” tool of the French Environment and Energy Management Agency, which provides emission factors and inventory datasets for organizations, was used to calculate the carbon footprint.

The results indicated that the mean daily carbon footprint was 1688.65 kgCO2eq, which translates to a mean of 9.28 kgCO2eq per patient. The majority of these emissions (78.4%) originated from patient and staff transportation (1324.76 kg CO2eq). The remainder came from energy consumption (6.8%), medicines and medical devices (12.3%), equipment (0.9%), and waste (1.6%). When minor interventions like intravitreal injections and anterior chamber lavage were included, medicines and medical devices (77%) emerged as the primary sources.

### So, what are healthcare professionals’ views on sustainability and the necessary precautions?

A study of New Zealand ophthalmologists’ views on sustainability found that a majority were positive about sustainable changes that could reduce costs and carbon footprints. However, 19% (9/47) of respondents stated that climate change was not caused by human behavior and did not need to be mitigated. More young ophthalmologists than those over 50 believed broad-based political action was necessary for sustainability [63]. Similarly, a study of 1300 US ophthalmologists and nurses revealed that 93% of respondents believed waste was produced in healthcare delivery and should be minimized, while 90% expressed concern about global warming. However, only 78% of respondents supported reusing materials, likely due to the risk of contamination [64].

### Barriers to sustainability measures

Sustainability appears straightforward to implement in ophthalmology practice, yet they encounter numerous systemic, institutional, and practical challenges. The most significant issue is the lack of awareness and data. Many ophthalmologists and healthcare institutions are unaware of the environmental impact of their practices [38, 39]. Furthermore, few studies assess the CO2eq emissions of ophthalmology practices. The Eyefficiency application, which stands out in this field, is a data collection and analysis tool that evaluates the efficiency of eye care services, particularly cataract surgery. However, it has the drawback that supply emissions are estimated solely based on supply costs and are greatly influenced by the lack of transparency further up the supply chain [65]. In addition, single-use instruments are preferred to reduce the risk of infection despite increasing waste. Although reuse may be possible in some cases, legislation might restrict it. Concerns about quality and patient safety also exist. Another issue is the absence of incentives and policy support for sustainable options. Furthermore, many ophthalmologists and nurses believe that workplace policies hinder sustainable practices [64]. These policies encompass hospital accreditation standards, infection control procedures, and regulatory requirements for utilizing medical devices or pharmaceuticals. While organizations such as the American Academy of Ophthalmologists and Royal Australian and New Zealand College of Ophthalmologists provide resources like consensus statements or practice guidelines to expand sustainability practices, it is clear that these stereotypical policies still need to be addressed [66]. Sustainable alternatives for devices and packaging remain relatively limited and not cost-effective. Sustainable technologies, such as digital patient records and low-energy diagnostic tools, are still not widely available in many clinics. To overcome these barriers, sustainable practices in ophthalmology must be encouraged and supported at the policy level.

### What actions can be taken in the future?

Carbon footprint assessments remain rare in ophthalmology. Promoting and ensuring access to these assessments can raise clinician awareness and enhance institutional policies. Future studies should examine energy use specific to ophthalmology and create more environmentally friendly practices. Teleophthalmology and AI-assisted examinations present promising solutions to alleviate clinic overcrowding and optimize resource usage. They should be expanded and developed further without compromising patient care. More studies are necessary to assess the environmental impact of single-use instruments and the safety and feasibility of reusable alternatives across various ophthalmology subspecialties. Finally, sustainability efforts must consider low- and middle-income countries’ realities. Research that considers local needs, resources, and cultural factors may help develop strategies that are more applicable on a global scale.

In conclusion, ophthalmologic care contributes significantly to the global carbon footprint because of its wide-ranging scope and specialized needs. Although much of the literature has focused primarily on operating rooms and surgical procedures, crucial steps can also be taken in outpatient clinic services. Among the measures that can be taken are redesigning outpatient clinics, using multipurpose tools and drops with appropriate disinfection and sterilization instead of single-use ones, reducing unnecessary referrals and visits, and integrating AI and telemedicine into ophthalmologic care.

Besides, when providing health care, we must also consider the public interest, which is inseparable from the interests of the physical environment in which we live. Reducing unnecessary costs and ensuring feasibility are essential for delivering quality health care. While striving to create “green clinics”, prioritizing cost-effective and high-impact modifications—such as redesigning outpatient clinics, using reusable and multi-use drugs and materials, and reducing unnecessary referrals and visits—will enhance the quality and efficiency of healthcare services.

## Data Availability

The datasets were generated from open-source databases. All data are available within the manuscript and its supplemental files.
